# How I manage a difficult intubation

**DOI:** 10.1186/s13054-019-2451-4

**Published:** 2019-05-16

**Authors:** Jonathan D. Casey, Matthew W. Semler, Kevin High, Wesley H. Self

**Affiliations:** 1 Division of Allergy, Pulmonary, and Critical Care Medicine, Vanderbilt University Medical Center, Nashville, TN USA; 2 Department of Emergency Medicine, Vanderbilt University Medical Center, Nashville, TN USA

In this article, we review our approach to a difficult intubation in a critically ill adult who is not in cardiac arrest and located in an emergency department (ED) or intensive care unit (ICU). Tracheal intubation may be difficult for either anatomical or physiological reasons. An anatomically difficult intubation (sometimes referred to as a “difficult airway”) involves challenges in viewing the vocal cords (difficult laryngoscopy) or passing a tube into the trachea (difficult endotracheal tube placement). A physiologically difficult intubation involves cardiopulmonary compromise, typically manifested as hypoxemia or hypotension.

## Standardization

In this article, we review our approach to a difficult intubation in a critically ill adult who is not in cardiac arrest and located in an emergency department (ED) or intensive care unit (ICU). Tracheal intubation may be difficult for either anatomical or physiological reasons. An anatomically difficult intubation (sometimes referred to as a “difficult airway”) involves challenges in viewing the vocal cords (difficult laryngoscopy) or passing a tube into the trachea (difficult endotracheal tube placement). A physiologically difficult intubation involves cardiopulmonary compromise, typically manifested as hypoxemia or hypotension.

Anatomical and physiological difficulties are often unable to be anticipated based on a pre-intubation assessment [1, 2]. Therefore, to be prepared for an unanticipated difficult intubation, we complete the following in a standardized fashion for all intubations [3]. We ensure that rescue devices (oropharyngeal airway, nasopharyngeal airway, bougie, laryngeal mask airway (LMA), cricothyrotomy equipment), phenylephrine, and intravenous crystalloid solutions are visible and immediately accessible. Two proceduralists participate in the intubation. The primary proceduralist stands at the head of the bed while the secondary proceduralist stands adjacent to the patient’s left ear. The primary proceduralist takes charge of pre-oxygenation, airway patency maneuvers, selecting medications, laryngoscopy, and endotracheal tube placement; if bag-mask ventilation is performed, the primary proceduralist is responsible for the mask seal in a two-person bag-mask technique. The secondary proceduralist takes charge of monitoring the vital signs, handing the primary proceduralist equipment, maintaining laryngeal position after external laryngeal manipulation by the primary proceduralist, and delivering ventilations during two-person bag-mask ventilation. We apply the *Vortex* [4] approach—once a single “best effort” has been made by the most experienced proceduralist available for any technique (e.g., tracheal intubation, LMA, bag-mask ventilation), the team moves to another technique with rapid progression to cricothyrotomy in the rare cases of “can’t intubate, can’t oxygenate.”

## Anatomically difficult intubation

We routinely assess for the following risk factors for an anatomically difficult intubation prior to the procedure: documentation of a prior difficult intubation, jaw immobility, neck immobility (when a cervical collar is not required), deformity of the face or neck, blood or vomit in the mouth, inability to visualize the uvula with mouth opening, and airway sounds suggesting an upper airway obstruction. Altered mental status and cervical immobilization with a hard collar preclude many critically ill patients from completing classic airway assessments, such as Mallampati scoring [2].

**Fig. 1:**
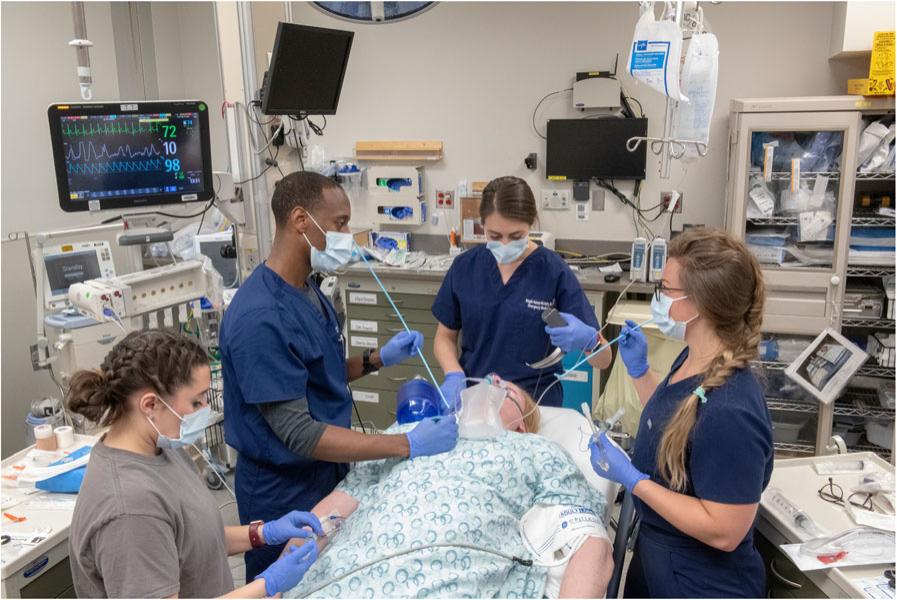
Our setup for an intubation with high-risk features for anatomical difficulty: the primary proceduralist at the head of the bed with a video laryngoscope in hand and a bag-valve mask immediately available; the secondary proceduralist to the patient’s left monitoring vital signs and with a bougie in hand and a laryngeal mask airway immediately available; and a tertiary proceduralist to the patient’s right with a scalpel and bougie prepared to perform open cricothyrotomy. Photograph by Lawrence B. Stack, MD. The photograph includes (clockwise from the head of the bed): Maglin Halsey, MD; Kaitlyn Works, MD; Joseph Sikon, MD; Lauren Nowaczyk, RN; Joshua Norman, MD

For patients with high-risk features for anatomical difficulty, we supplement our standard procedure with the following: (1) video laryngoscopy with a non-hyperangulated blade for the first attempt at laryngoscopy [5], (2) bougie use for the first attempt at intubating the trachea [6], and (3) addition of a third proceduralist, located on the patient’s right side, who is prepared to perform open cricothyrotomy [7] (Fig. 1). Our preferred approach for cricothyrotomy is a bougie-assisted open cricothyrotomy consisting of a vertical incision with a #10 scalpel, insertion of a bougie into the trachea, and placement of a cuffed 6.0 ETT over the bougie [7, 8].

## Physiologically difficult intubation

Patients with severe chronic lung disease, acute hypoxemic respiratory failure, or an SpO_2_ < 100% after pre-oxygenation are at increased risk for hypoxemia during intubation [9]. Providing positive pressure ventilation for these high-risk patients during pre-oxygenation and between induction and laryngoscopy can help prevent hypoxemia [10, 11]. The primary concern regarding positive pressure ventilation in this setting is aspiration. For patients at high risk for hypoxemia and low risk for aspiration (e.g., those without vomiting, hematemesis, or hemoptysis), we pre-oxygenate with non-invasive bilevel positive airway pressure (BiPAP) ventilation with 100% FiO_2_ for 5 min whenever feasible [10]. For patients at high risk for hypoxemia and high risk for aspiration, we pre-oxygenate with 60 l per minute of 100% FiO_2_ via high-flow nasal cannula or with supplemental oxygen via standard face mask and nasal cannula [12, 13]. The recent PreVent trial found that positive pressure bag-mask ventilation between induction and laryngoscopy reduced severe hypoxemia during tracheal intubation in the ICU [11]. The PreVent trial excluded patients with very high risk for aspiration (e.g., vomiting, hematemesis, hemoptysis). Therefore, for patients with very high risk of aspiration, we provide supplemental oxygen alone without positive pressure after induction, whereas for patients at high risk for hypoxemia and without high-risk features for aspiration, we provide positive pressure ventilation with either BiPAP or bag-mask ventilation between induction and laryngoscopy.

Severe hypotension during intubation can lead to cardiac arrest and death. Mechanisms that contribute to peri-intubation hypotension include vasodilation from induction medications, decreased sympathetic tone from sedation, and decreased venous return from increased intrathoracic pressure with positive pressure ventilation. We attempt to reverse pre-existing hypotension prior to intubation by administering blood products for hemorrhagic shock and intravenous fluids and vasopressors for distributive shock. Additionally, we commonly administer phenylephrine 100 mcg by IV push to treat peri-intubation hypotension. For patients at risk for hypotension during intubation [14], we use ketamine for induction and avoid other agents more likely to contribute to hypotension, understanding that data supporting this practice are incomplete [15]. For patients without pre-procedure hypotension, whether prophylactically administering an intravenous fluid bolus or a vasopressor prior to induction prevents cardiovascular collapse remains the subject of ongoing research (NCT03026777). Currently, we do not routinely administer prophylactic fluids or vasopressors prior to induction in patients who are not hypotensive. Immediately after intubation, we set the mechanical ventilator to preserve ventilatory compensation for metabolic acidosis and avoid tidal volumes > 6 ml/kg of ideal body weight.

## Conclusion

Difficult intubations cannot always be predicted. Therefore, our approach involves standardized preparation and execution of each intubation in a manner that can address anatomical or physiological difficulties as they are encountered. We incorporate new techniques into this paradigm as emerging literature and our experience support such incorporation. Recent advances in our approach include the use of video laryngoscopy and a bougie on the first attempt for intubations with anticipated anatomical difficulties, ketamine as an induction agent in hypotensive patients, and use of positive pressure ventilation for pre-oxygenation and between induction and laryngoscopy for patients at high risk for hypoxemia and low risk for aspiration.
